# Abnormal bile acid-microbiota crosstalk promotes the development of hepatocellular carcinoma

**DOI:** 10.1007/s12072-022-10299-7

**Published:** 2022-02-24

**Authors:** Rui Shen, Lixin Ke, Qiao Li, Xi Dang, Shunli Shen, Jianming Shen, Shaoqiang Li, Lijian Liang, Baogang Peng, Ming Kuang, Yi Ma, Zhonghan Yang, Yunpeng Hua

**Affiliations:** 1 Hepatobiliary and Pancreatic Surgery Center, First Affiliated Hospital, Sun Yat-Sen University, 58 Zhongshan 2nd Road, Guangzhou, 510080 People’s Republic of China; 2 Department of Liver Surgery, Guangdong Provincial People’s Hospital, Guangzhou, People’s Republic of China; 3 Department of Organ Transplantation, First Affiliated Hospital, Sun Yat-Sen University, Guangzhou, People’s Republic of China; 4 Department of Biochemistry, Zhongshan School of Medicine, Sun Yat-Sen University, Guangzhou, Guangdong People’s Republic of China

**Keywords:** Primary bile acids, Secondary bile acids, Gut microbiota, Bile salt hydrolase, Glyco-deoxycholic acid, Bifidobacteriales, Lactobacillales, Bacteroidales, Clostridiales, Tumorigenesis

## Abstract

**Background:**

Gut microbiota and microbe-derived metabolites are involved in the development of HCC. Bile acids (BAs) are the most important gut microbiota-modulated endogenous signaling molecules.

**Methods:**

We tested serum bile acid levels and gut microbiome compositions in patients with HCC, chemical-induced HCC mouse models (DEN-HCC mice) and mouse orthotopic implanted liver tumor models with vancomycin treatment (vancomycin-treated mice). Then, we screened an important kind of HCC-related BAs, and verified its effect on the growth of HCC in vivo and in vitro.

**Results:**

We found that the remarkably decreasing percentages of serum secondary BAs in the total bile acids of patients and DEN-HCC mice, especially, conjugated deoxycholic acids (DCA). The relative abundance of the bile salt hydrolase (BSH)-rich bacteria (Bifidobacteriales, Lactobacillales, Bacteroidales, and Clostridiales) was decreased in the feces of patients and DEN-HCC mice. Then, in vancomycin-treated mice, vancomycin treatment induced a reduction in the BSH-rich bacteria and promoted the growth of liver tumors. Similarly, the percentage of conjugated DCA after vancomycin treatment was significantly declined. We used a kind of conjugated DCA, Glyco-deoxycholic acid (GDCA), and found that GDCA remarkably inhibited the growth of HCC in vivo and in vitro.

**Conclusions:**

We conclude that the remarkably decreasing percentages of serum conjugated DCA may be closely associated with HCC, which may be induced by the reducing gut BSH-rich bacteria. The mechanisms may be correlated with conjugated DCA directly inhibiting the growth and migration of HCC cells.

**Supplementary Information:**

The online version contains supplementary material available at 10.1007/s12072-022-10299-7.

## Introduction

Globally, hepatocellular carcinoma (HCC) is still the fourth most common cause of cancer-related deaths, with nearly 800,000 new cases annually, despite many recent advances in the diagnosis and treatment of HCC [1–4]. Therefore, it is urgent to understand the mechanisms of HCC occurrence and progression and to find novel approaches to predict or treat HCC. It is well known that hepatocarcinogenesis is closely related to chronic liver injury resulting from hepatic inflammation, which is mainly attributed to hepatitis B virus infection. Furthermore, increasing evidence also suggests that other intrahepatic and systemic factors likely play significant roles in the process of carcinogenesis and progression, such as the gut microbiota, microbe-derived metabolites and bile acids (BAs) [5–8].

The gut microbiota has been thought to play relevant roles in physiological conditions of human health, such as digestion, vitamin B synthesis, immunomodulation, and the promotion of angiogenesis and nerve function [9]. Recently, increased interest has also focused on the specific role of the intestinal microbiota in various metabolic diseases, including alcoholic liver disease, nonalcoholic fatty liver disease, liver cirrhosis, and even HCC, because the liver is the first organ to be exposed to gut-derived toxic factors through the portal vein [10]. A study showed that fecal microbial diversity was increased from cirrhosis to early HCC with cirrhosis [11]. The gut microbiota can promote the development of HCC through the gut-liver axis in animal models [12, 13], and probiotics can inhibit the growth and tumor angiogenesis of HCC by regulating the gut bacteria of mice [14]. Bifidobacterium enhances antitumor immunity by enhancing the function of dendritic cells and efficacy of anti-PD-L1 therapy [15]. However, until now, the mechanisms by which the gut microbiota promotes HCC have not yet been clarified.

It is well known that the gut microbiota can convert the primary BAs chenodeoxycholic acid (CDCA) and cholic acid (CA) into the secondary BAs lithocholic acid (LCA), deoxycholic acid (DCA) and ursodeoxycholic acid (UDCA) through deconjugation by bile salt hydrolase (BSH) and downstream modifications by 7-alpha-dehydroxylase or 7-alpha-hydroxysteroid dehydrogenase (HSDH) [16, 17]. As microbe-derived metabolites, BAs are involved in the induction of hepatocellular injury, in addition to facilitating lipid absorption [5]. In general, the hydrophobic bile acids LCA, DCA, and CDCA are cytotoxic, and the hydrophilic bile acid UDCA and its derivative taurourso-deoxycholic acid (TUDCA) are cytoprotective [18]. In addition, BAs also have direct or indirect antimicrobial effects to modulate the constitution of the microbiota, which in turn influences the size and composition of the BA pool [19]. Recently, accumulating evidence has demonstrated that bile acid–microbiota crosstalk plays a crucial role in gastrointestinal carcinogenesis [20]. Yoshimoto et al. also showed that the gut bacterial metabolite DCA promoted the development of obesity-associated HCC, which was induced with 7,12­dimethylbenzanthracene (DMBA) in a mouse model [6]. In contrast, the hydrophilic bile acid TUDCA diminished liver overgrowth and tumorigenesis in mice [21]. Xie et al. showed that the levels of hydrophobic BAs in plasma and liver were substantially increased in a nonalcoholic steatohepatitis-hepatocellular carcinoma (NASH-HCC) mouse model, including DCA, tauro-cholate acid (TCA), tauro-chenodeoxycholate acid (TCDCA), and tauro-lithocholate acid (TLCA). Furthermore, 2% cholestyramine feeding significantly prevented HCC development by enhancing the intestinal excretion of hydrophobic BAs [22]. However, the characteristics of bile acids in HCC patients have not yet been reported, and it remains unclear how the gut microbiota influences the levels and species of bile acids in patients with HCC. Therefore, we hypothesized that the gut microbiota-bile acid axis was closely associated with the development of HCC and that it was very valuable to probe the mechanisms of and find novel targets for the diagnosis and treatment of HCC.

In this study, we revealed the unique gut microbial spectrum and bile acid spectrum of patients and mice with HCC, explored the correlation between host microbes and bile acids, and further confirmed the protective role of hydrophilic conjugated secondary bile acids on HCC.

## Materials and methods

### Human subjects

The study was approved by the Ethics Review Committee of the First Affiliated Hospital of Sun Yat-sen University. All participants were recruited from the Department of Hepatic Surgery at Sun Yat-sen University First Affiliated Hospital, which included 20 individuals with hepatitis B virus (HBV)-related HCC and 15 healthy controls. Written informed consent was obtained from all participants. None of the individuals were positive for hepatitis C virus (HCV), consumed excessive alcohol, or received chemotherapy before sampling. The main clinical characteristics of human subjects were summarized in Table 1. In addition, 11 patients had a single tumor with a median diameter of 6.2 cm (range: 3.2–12.5 cm), and 9 patients had multiple tumors with a median diameter of 5.1 cm (range: 2.8–20.3 cm). According to the BCLC staging system, the number of stage A, B, and C patients was 12 (60%), 5 (25%), and 3 (15%), respectively.

**Table 1:** Patient characteristics for serum bile acid analysis

	Group		
	Healthy control (*N* = 15)	HCC (*N* = 20)	*p* value
Age(year)	27.73 ± 10.75	51.30 ± 10.18	*p* < 0.001
Gender(F/M)	F12/M3	F16/M4	*p* > 0.05
BMI(kg/m^2^)	19.8 ± 2.83	21.2 ± 4.75	*p* > 0.05
AFP (μg/L)			
≤ 20	15 (100%)	5 (25%)	*p* < 0.001
> 20	0 (0%)	15 (75%)	
ALT	18.73 ± 5.96	86.85 ± 125.85	*p* = 0.026
AST	19.53 ± 4.10	76.60 ± 58.92	*p* < 0.001
Albumin	40.21 ± 3.56	37.09 ± 4.05	*p* = 0.024
Child-Paugh			
A	15 (100%)	19 (95%)	–
B	0 (0%)	1 (5%)	–

*HCC* hepatocellular carcinoma; *ALT* alanine aminotransferase; *AST* aspartate aminotransferase; *AFP* alpha fetoprotein; *F* female; *M* male

All blood samples were set at room temperature for 30 min and were then centrifuged at 3000*g* for 20 min to obtain the serum. Fecal samples were collected on the same day, snap frozen in dry ice, and stored at −80 ℃ until analysis.

### Cell culture

The H22 mouse HCC cell line, SUN-449 and HepG2 human HCC cell lines, and LO2 human hepatocyte cell line were purchased from the Shanghai Cell Collection (Shanghai, China) and maintained in Dulbecco’s modified Eagle’s medium (DMEM) with 10% fetal bovine serum (FBS) (Gibco by Life Technologies, Bleiswijk, the Netherlands). Cells were cultured in a cell incubator with 5% CO_2_ at 37 °C.

### Diethylnitrosamine (DEN) and carbon tetrachloride (CCl4)-induced HCC C57BL/6J mouse model

Male C57BL/6 mice were purchased from Vital River Laboratories (Beijing, China). C57BL/6J mice (6 weeks old) were divided into the following two groups (*n* = 10 in each group): (1) HCC group, HCC was induced by the intraperitoneal (i.p.) injection of diethylnitrosamine (DEN) (100 mg/kg) at 6 weeks of age followed by 6–12 biweekly injections of carbon tetrachloride (0.5 ml/kg i.p. dissolved in corn oil) unless stated otherwise; (2) control group, i.p. with corn oil as vehicle, double distilled (Ddwater) (25 mg/kg i.p.) was given at day 15 postpartum, and 6–12 weekly injections of corn oil (0.5 ml/kg i.p.) [12]. The mice were sacrificed at week 32, and the size of the liver tumors was measured. Blood serum samples were collected for BA assessment. Stool samples were collected for 16S RNA analysis. All samples were stored at −80 ℃ until analysis.

### Orthotopic C57BL/6 mouse hepatic tumor model with gut microflora dysbiosis

Male C57BL/6 mice (6–8 weeks old) were randomly divided into two groups (*n* = 5 each group). Gut bacterial dysbiosis in the vancomycin group was induced using vancomycin (mainly sterilized gram-positive bacteria, 500 mg/l) in drinking water for 4 weeks. Mice in the control group drank sterile water directly. H22 mouse HCC cells (1 × 10^6^ in 200 µl DMEM) were injected subcutaneously into the flanks of C57BL/6 mice to generate implanted tumors. After 2 weeks, the subcutaneous tumors were resected and diced into 1 mm^3^ cubes, which were then implanted in the left lobe of the liver to make orthotopic transplantation tumors in C57BL/6 mice in the vancomycin group and control group. All of the mice were killed after 2 weeks, and the size of the liver tumors was measured. Blood serum samples were collected for BA assessment. Stool samples were collected for 16S RNA analysis. All samples were stored at −80 ℃ until analysis. All studies were conducted with the approval of the Institutional Animal Care and Use Committee (IACUC) of the First Affiliated Hospital of Sun Yat-sen University.

### Subcutaneous tumor transplant model with GDCA treatment

Eight-week-old, athymic BALB/c nu/nu female mice were purchased from Gempharmatech Co., Ltd (Nanjing, China). Mice were randomly divided into two groups: GDCA group (*n* = 5) and control group (*n* = 5). Nude mice were subcutaneously transplanted with SUN-449 cells (5 × 10^6^ cells in 150 μl PBS). On day 10 after cell injection, mice were treated with GDCA (200 mg/kg every day) or PBS (equal amount) by gastric gavage. Treatments were maintained for one month. Then, mice were sacrificed, and tumor tissues were removed, weighed, and photographed. Tumor volumes were determined by measuring length (*l*) and width (*w*) and calculating volume (*V* = 0.5 × l × *w*^2^) at the indicated time points.

### Bile acid analysis

Serum samples were prepared by precipitation. In addition, 50 μl of sample was transferred to an EP tube. After the addition of 200 μl of extraction solvent (acetonitrile-methanol, 1:1, containing 0.1% formic acid and 312.5 nmol/l internal standard), the samples were vortexed for 30 s, sonicated for 10 min in an ice-water bath, incubated at −40 °C for 1 h and centrifuged at 12,000 rpm and 4 °C for 15 min. The clear supernatants were transferred to an autosampler vial for ultra-high performance liquid chromatography tandem mass spectrometry (UHPLC-MS/MS) analysis.

### 16S rRNA sequencing and analysis

Human and mouse stool sample collection was described previously. Briefly, the sample was divided into five aliquots of 200 mg and immediately stored at −80 °C. Total DNA in feces was isolated using a DNA extraction kit (Tiangen, China). The V3-V4 hypervariable regions of the bacterial 16S rRNA gene were amplified with primers 338F (5′-ACTCCTACGGGAGGCAGCAG-3′) and 806R (5′-GGACTACHVGGGTWTCTAAT-3′). PCR products were purified using the GeneJET Gel Extraction Kit (Thermo Scientific). Sequencing libraries were generated using an Illumina TruSeq DNA PCR-Free Library Preparation Kit (Illumina, USA) following the manufacturer’s recommendations, and index codes were added. The sequencing was performed by an Illumina HiSeq platform (Novogene Bioinformatics Technology Co., Ltd.). Sequence analysis was performed by Uparse software (Uparse v7.0.1001, http://drive5.com/uparse/). Sequences with ≥ 97% similarity were assigned to the same OTUs. The obtained OTU sequences were grouped at the phylum and order levels.

### Bacterial diversity and taxonomic analysis

Alpha diversity was applied to analyze the complexity of species diversity for a sample. All of these indices reflecting our samples were calculated with QIIME (Version 1.7.0) and displayed with R software (Version 2.15.3). Beta diversity analysis was used to evaluate differences in samples with regards to species complexity. Beta diversity on both weighted and unweighted UniFrac were calculated by QIIME software (Version 1.7.0). Principal coordinate analysis (PCoA) was performed with the WGCNA package, stat packages and ggplot2 package in R software (Version 2.15.3).

### Plate cloning formation experiment

SUN-449 and HepG2 human HCC cells and LO2 human hepatocyte cells were harvested and diluted with DMEM or RPMI 1640 medium. Then, SUN-449 and HepG2 cells were seeded at 1000 cells/well in a 6-well plate, while LO2 cells were seeded at 2000 cells/well in a 6-well plate. The treatment groups were treated with GDCA (0.5 mM), and the control groups were treated with PBS. The culture medium was changed every 3 days, and the cells were cultured for 14 days. Then, the cells were fixed with 4% formaldehyde and stained with crystal violet staining solution for 15 min. Next, the cells were air-dried at room temperature, and the plates were imaged. The number of colonies in each well was manually counted. Clone formation rate = clone number/number of inoculated cells × 100% [23].

### Cell counting kit-8 (CCK-8)

SUN-449 cells, HepG2 HCC cells and LO2 human hepatocytes were treated with GDCA (0.5 mM) or PBS for the cell growth test, which was detected by a Cell Counting Kit-8 (CCK-8) (Dojindo, Japan) according to the manufacturer’s protocol. The cells (2000 cells/well) were cultivated in 96-well plates for 24, 48, 72, 96, and 120 h, incubated with 10 μl of CCK-8 plus 100 μl of DMEM for 2 h, and finally placed in a microplate reader (BioTek Synergy2, Winooski, VT, USA) to measure the absorbance at 450 nm [24].

### Wound haling and transwell assays

The cell migration of SUN-449 and HepG2 HCC cells was evaluated using wound healing assays and Transwell assays. The treatment groups were treated with GDCA (0.5 mM), and the control groups were treated with PBS. Wound healing assays were performed as previously described by Liang et al. [25]. Briefly, the cells were seeded at 1 × 10^4^ cells/well in 6 cm dishes. After the cells formed a monolayer, a scratch wound was made with the tip of a 200-μl pipette tip. Photographs were taken at 0, 8, 16 and 24 h after wounding. Migration distances were measured using ImageJ software (National Institutes of Health, Bethesda, MD, USA).

Transwell assays were performed using Corning^®^ Transwell^®^ polycarbonate membrane cell culture inserts (pore size, 8.0 μm). A 200 μl aliquot of a 20,000 cells/ml suspension (SUN-449 and HepG2 HCC cells) was resuspended in serum-free medium supplemented with GDCA and seeded into the upper chamber of the polycarbonate membrane. Subsequently, 600 μl of medium containing 15% fetal bovine serum (FBS) was added to the lower well of the migration plate. After incubating for 24 h at 37 °C, cells in the upper layer of the membrane were scraped, and cells in the lower layer were stained with crystal violet staining solution. Then, the cells were photographed and counted under a phase contrast microscope [26].

### Cell apoptosis

SUN-449 and HepG2 HCC cells were treated with GDCA (0.5 mM) or PBS for cell apoptosis, which was detected 24 h after treatment using an Annexin V FITC apoptosis detection kit according to the manufacturer’s protocol (Dojindo, Japan). The cell apoptosis rate was calculated as follows: cell apoptosis rate (%) = (early apoptotic cells + advanced apoptotic cells)/total cell number × 100% [27].

### Additional statistical analysis

Student’s *t*-tests or Mann–Whitney *U* tests with two-tailed distribution were performed to examine significant differences between the two experimental groups. A *p* value < 0.05 was considered to be significant. All analyses were performed with GraphPad Prism 7.0 and SPSS 25.0 software.

## Results

### Serological bile acids in HCC patients and mice

**Fig. 1:**
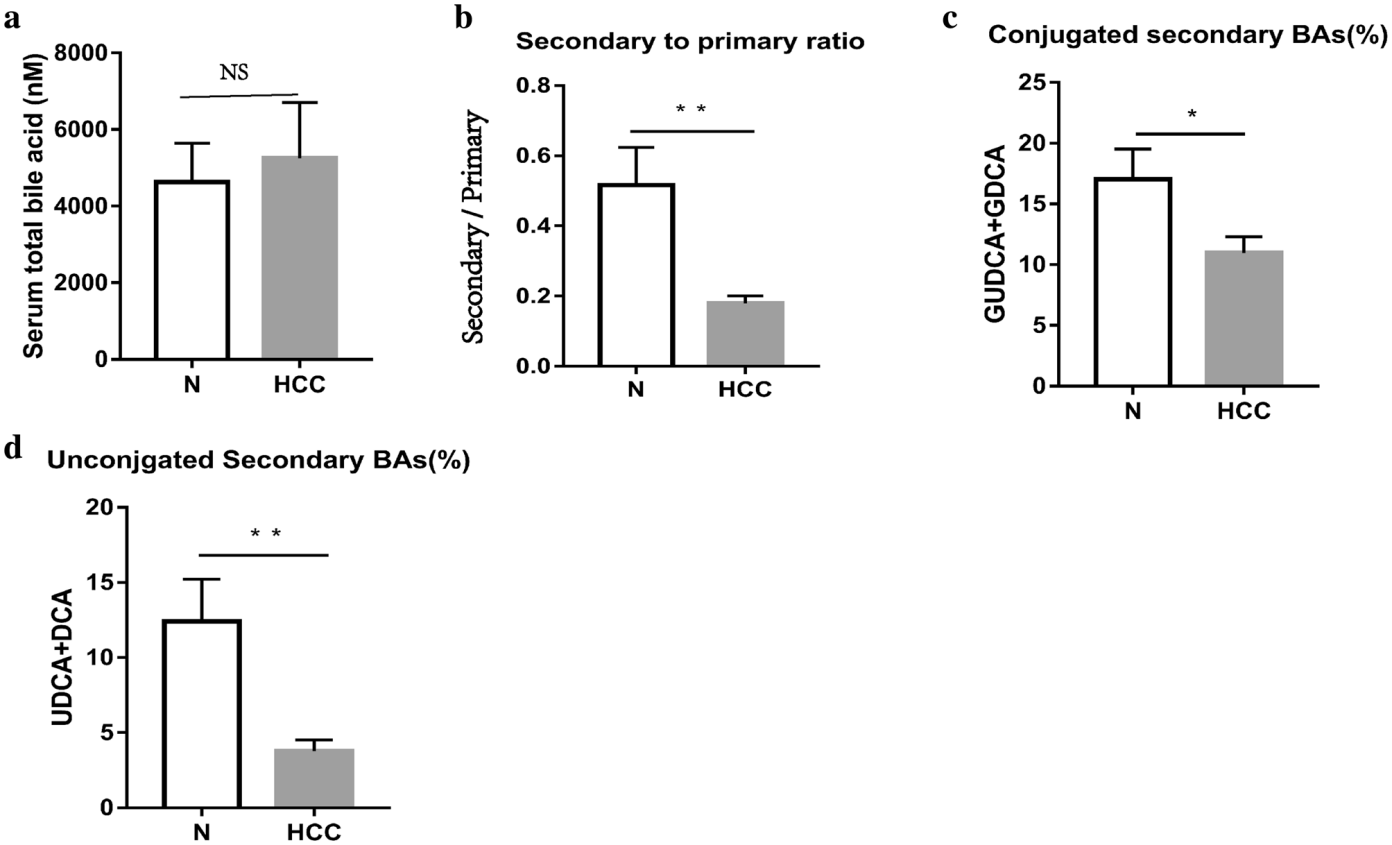
Serum bile acids in patients with HCC and healthy controls. **a** Plotted in the bar graph are total serum bile acids (MEAN ± SEM) in the serum of patients with HCC and healthy controls. **b** Ratio of secondary bile acids (DCA, GDCA, GUDCA, and UDCA) to Primary bile acids (CA, GCA, GCDCA, and CDCA). **c** Percent of conjugated secondary bile acids (GDCA and GUDCA) in the serum of patients with HCC and healthy controls. **d** Percent of unconjugated secondary bile acids (DCA and UDCA) in the serum of patients with HCC and healthy controls. *HCC* hepatocellular carcinoma; *N* healthy controls. *p < 0.05, **p < 0.01, ***p < 0.001

The levels of serum total BAs in the HCC group (5251 ± 1460 nM) were higher than those in the healthy controls (4626 ± 1015 nM), but this difference did not reach statistical significance (*p* = 0.736) (Fig. 1a). We found there was a significantly lower ratio of secondary BAs to primary BAs in the serum of the HCC group (0.18 ± 0.02) than in the healthy control group (0.52 ± 0.11) (*p* = 0.008) (Fig. 1b). Specifically, the percentages of conjugated (HCC group 11.0% ± 1.3%, healthy control group 17.1% ± 2.4%) (*p* = 0.028) and unconjugated secondary BAs (HCC group 3.8% ± 0.7%, healthy control group 12.4% ± 2.8%) (*p* = 0.009) in the total BAs were both significantly reduced in the HCC group (Fig. 1c, d).

**Fig. 2:**
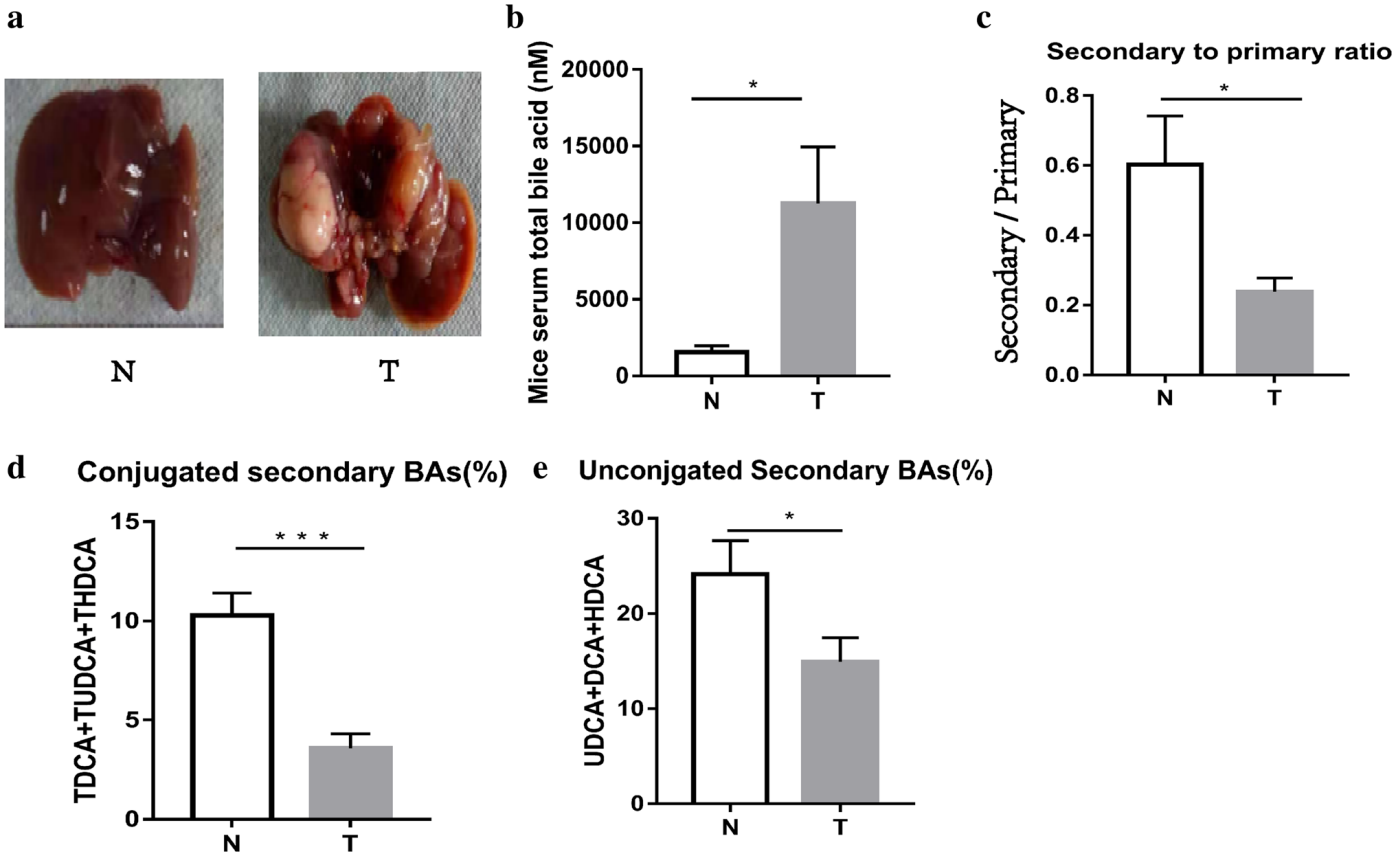
Serum bile acids in patients with chemical-induced mice and normal control mice. **a** Liver images from chemical-induced mice and normal control mice. **b** Plotted in the bar graph are Mice total serum bile acids (MEAN ± SEM). **c** Ratio of secondary bile acids (DCA, TDCA, TUDCA, UDCA, HDCA and THDCA) to primary bile acids (CA, TCA, TCDCA, CDCA, α-MCA, β-MCA, Tα-MCA, Tβ-MCA). **d** Percent of conjugated secondary bile acids (TDCA, TUDCA, and THDCA) in the serum of chemical-induced mice with HCC and normal control mice. **e** Percent of unconjugated secondary bile acids (DCA, UDCA, and HDCA) in the serum of chemical-induced mice with HCC and normal control mice. *T* Chemical-induced mice; *N* normal control mice; *p < 0.05, **p < 0.01, ***p < 0.001

To investigate the percentage of secondary BAs in the total BAs was decreased in HCC patients, we generated mouse liver cancer models through the combinative induction of diethylnitrosamine (DEN) and hepatotoxin carbon tetrachloride (CCl4) (Fig. 2a). We found that the level of serum total BAs was significantly increased in the DEN-HCC mouse group (11,244 ± 3690 nM) compared to that in the control mouse group (1556 ± 407 nM) (*p* < 0.001) (Fig. 2b). The ratio of secondary BAs to primary BAs was also remarkably reduced in the DEN-HCC mouse group (DEN-HCC mouse group 0.24 ± 0.04, control mouse group 0.60 ± 0.14) (*p* = 0.029) (Fig. 2c). Compared with those of the normal control mouse group, the percentages of conjugated (DEN-HCC mouse group 3.6% ± 0.7%, control mouse group 10.3% ± 1.1%) (*p* < 0.001) and unconjugated secondary BAs (DEN-HCC mouse group 14.9% ± 2.5%, control mouse group 24.1% ± 3.5%) (*p* = 0.048) of the DEN-HCC mouse group were also decreased (Fig. 2d, e).

### Characterization of gut microbiome compositional profiles in HCC patients and mice

**Fig. 3:**
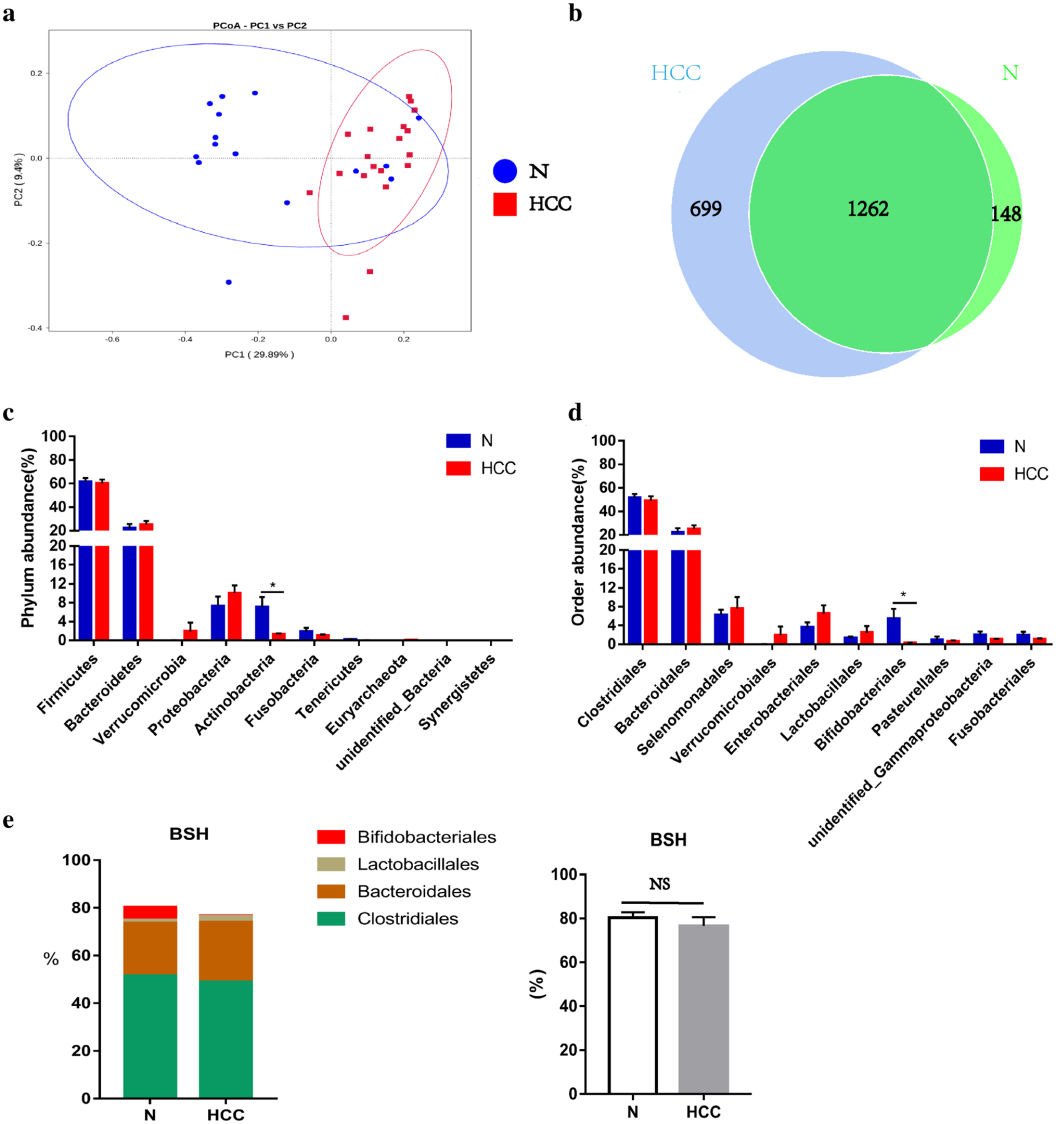
System composition spectrum of gut microbiome in HCC and healthy controls. Bile acid biosynthesis, transport and metabolism. **a** Principal Co-ordinates Analysis (PCoA) of bacterial beta diversity based on the unweighted UniFrac distances. Each node represents each sample; HCC and N subjects are colored in red and blue, respectively. **b** A Venn diagram displaying the overlaps between groups. **c** Relative abundance of the top 10 microbiota at the Phylum level in HCC and N. **d** Relative abundance of the top ten microbiota at the order level in HCC and N. **e**, **f** BSH include species in order. *HCC* hepatocellular carcinoma; *N* healthy controls; *p < 0.05; **p < 0.01, ***p < 0.001

It is well known that the gut microbiota can affect the metabolism of bile acids and change the composition of bile acids [8]. To display microbiome β-diversity, we used principal coordinate analysis (PCoA) coupled with unweighted UniFrac distances and found a clear separation between HCC patients and healthy controls (Fig. 3a). Moreover, to display the overlaps between two groups, we used a Venn diagram and observed that 1262 of the 2109 OTUs were shared between the 2 groups (Fig. 3b). We found 699 of 1961 OTUs were unique to HCC patients, while only 148 of 1410 OTUs were unique to healthy persons (Fig. 3b).

Among the bacterial compositions, the bacterial phyla Bacteroidetes, Firmicutes, Proteobacteria, and Actinobacteria were the most abundant bacteria. Compared with healthy controls, Actinobacteria was significantly decreased in HCC patients (*p* = 0.02). In addition, Bacteroidales, Lactobacillales, Selenomonadales, Verrucomicrobiales, and Enterobacteriales were increased in HCC, while Clostridiales, Fusobacteriales, Pasteurellales, and Burkholderiales were decreased in HCC, but these differences between them were not statistically significant (Fig. 3c). At the order level, probiotic Bifidobacteriales, belonging to the phylum Actinobacteria, was significantly decreased in HCC patients (*p* = 0.026) (Fig. 3d). Since the production of secondary bile acids requires the participation of BSH enzymes from Bifidobacteriales, Lactobacillales, Bacteroidales, and Clostridiales [18], we checked the abundance of BSH-rich bacteria in HCC patients (76.6% ± 4.0%) and observed that it was lower than that in healthy controls (80.3% ± 2.5%); however, their difference did not reach statistical significance (*p* = 0.462, Fig. 3e).

**Fig. 4:**
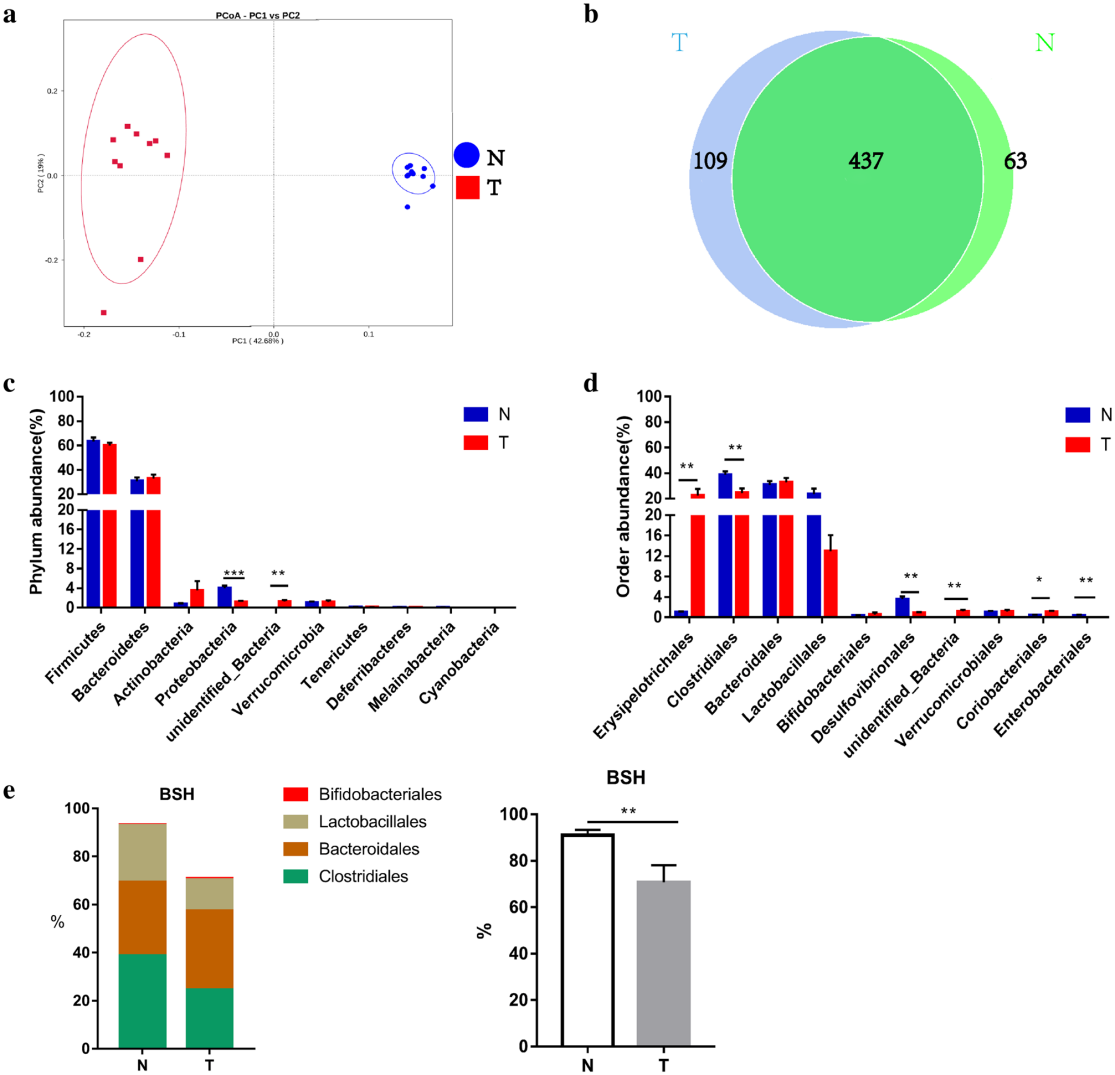
System composition spectrum of gut microbiome in DEN-induced HCC mice. **a** Principal Co-ordinates Analysis (PCoA) of bacterial beta diversity based on the unweighted UniFrac distances. Each node represents each sample. T and N subjects are colored in red and blue, respectively. **b** Venn diagram of OTUs in two groups. **c** Relative abundance of the top ten microbiota at the phylum level in two groups. **d** Relative abundance of the top microbiota at the order level in two groups. **e**, **f** BSH include species in order. *T* Chemical-induced mice; *N* normal control mice; *p < 0.05; **p < 0.01, ***p < 0.001

To investigate the HCC-related changes in the gut microbiome, we collected fecal samples from mice with DEN-induced HCC and controls. After using PCoA to display microbiome β-diversity, we found two distinct enterotypes between the two groups (Fig. 4a). Furthermore, a Venn diagram showed that 437 of the 609 OTUs were shared between the 2 groups. Notably, 109 of 546 OTUs were unique to mice with HCC, while only 63 of 500 OTUs were unique to control mice (Fig. 4b).

In addition, the bacterial phyla Bacteroidetes, Firmicutes, Proteobacteria, and Actinobacteria were still the most abundant bacteria in the two groups (Fig. 4c). Compared with control mice, phylum unidentified Bacteria were significantly increased in HCC (*p* = 0.004), and Proteobacteria were significantly decreased in HCC (*p* < 0.001) (Fig. 4c). At the order level, Erysipelotrichales (*p* = 0.002), unidentified Bacteria (*p* = 0.006), and Coriobacteriales (*p* = 0.02), were remarkably increased in mice with HCC, while Clostridiales (*p* = 0.005), Desulfovibrionales (*p* < 0.001), and Enterobacteriales (*p* = 0.007) were significantly decreased in HCC (Fig. 4d). We also found that the abundance of BSH-rich bacteria in DEN-induced HCC mice (70.7% ± 6.5%) was markedly lower than that in normal control mice (91.0% ± 0.6%) (*p* = 0.007) (Fig. 4e).

### Antibiotic vancomycin decreased the abundance of BSH-rich bacteria, lowered the levels of secondary BAs, and induced tumor growth

**Fig. 5:**
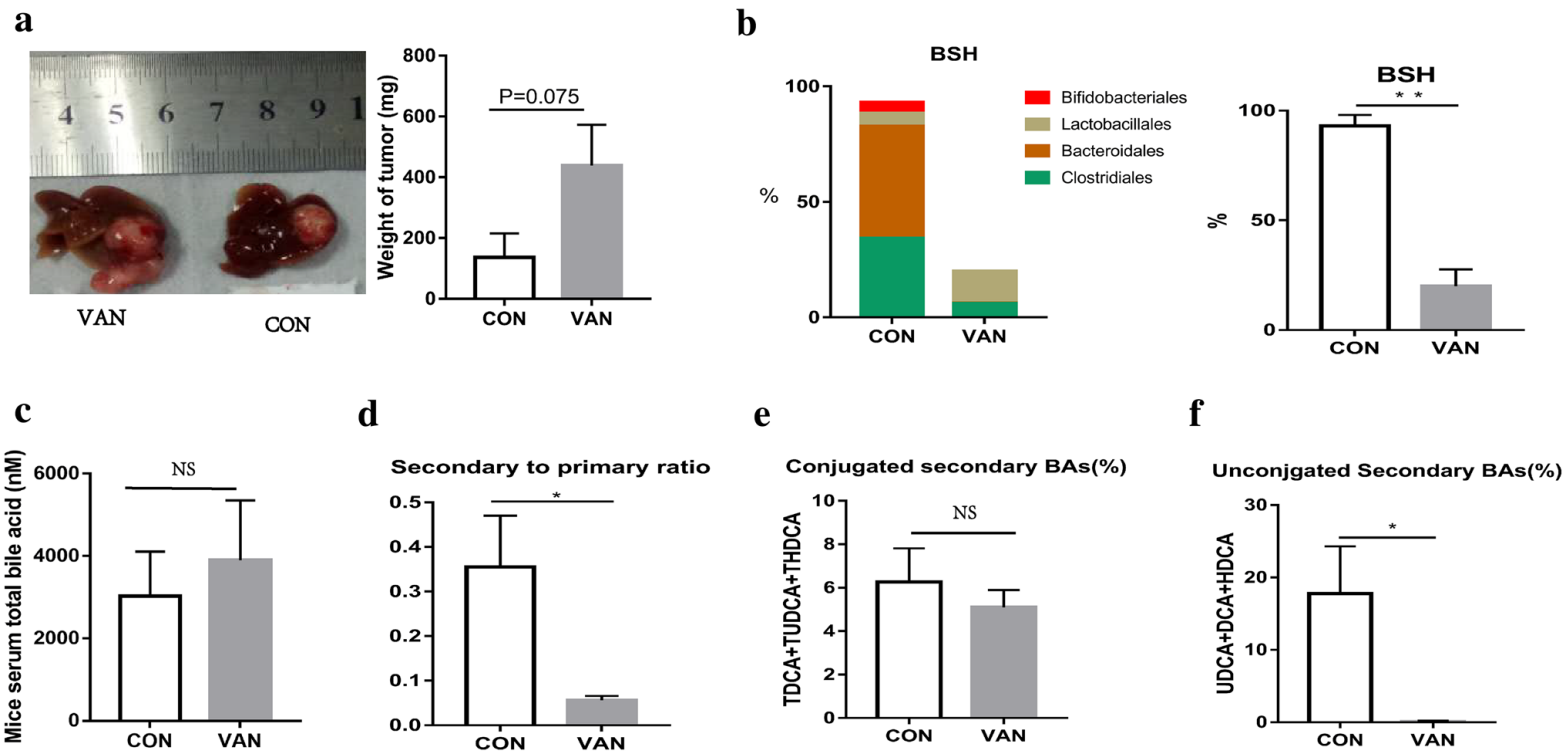
The decrease of BSH include species after vancomycin treatment leads to a decrease in secondary BAs, which promotes the development of HCC. **a** Images of tumors from each group and tumor weight at the time of sacrifice. **b**, **c** BSH include species in order. **d** Plotted in the bar graph are Mice total serum bile acids (mean ± SEM). **e** Ratio of secondary bile acids (DCA, TDCA, TUDCA, UDCA, HDCA and THDCA) to Primary bile acids (CA, TCA, TCDCA, CDCA,α-MCA,β-MCA, Tα-MCA and Tβ-MCA). **f** Conjugated secondary bile acids (TDCA, TUDCA, and THDCA) in the serum of vancomycin treatment group mice and control group mice. **g** Unconjugated secondary bile acids (DCA, UDCA, and HDCA) in the serum of vancomycin treatment group mice and control group mice. *VAN* vancomycin treatment group mice; *CON* control group mice; *p < 0.05; **p < 0.01, ***p < 0.001

To further confirm our hypothesis that the decrease in BSH-rich bacteria is involved in the development of HCC through downregulating the levels of secondary BAs, we used vancomycin to treat C57BL/6 mice and then generated orthotopic implanted liver tumor models. We found that the tumor weight in the vancomycin treatment group was higher than that in the control group (*p* = 0.075, Fig. 5a). Furthermore, we used 16S rDNA to analyze the gut microbiota between the two groups and found that the abundance of BSH-rich bacteria in the vancomycin treatment group (20.0% ± 3.4%) was significantly lower than that in the control group (93.0% ± 2.2%) (*p* = 0.009) (Fig. 5b). To further observe the effect of vancomycin treatment on serum bile acids, we found that the concentration of serum total BAs in the vancomycin treatment group (3895 ± 1495 nM) was higher than that in the control group (3026 ± 1079 nM), but the difference was not statistically significant (*p* = 0.644, Fig. 5c). Interestingly, the ratio of secondary BAs to primary BAs of vancomycin treatment group (0.05 ± 0.01) was significantly lower than that of the control group (0.35 ± 0.11) (*p* = 0.032) (Fig. 5d). However, the percentage of conjugated secondary BAs in vancomycin treatment group (5.1% ± 1.0%) was significantly lower than that in control group (6.3 ± 1.5%) (*p* = 0.522) (Fig. 5e), though the percentage of unconjugated secondary BAs was significantly reduced in vancomycin treatment group (0.1% ± 0.1%) compared with control group (17.8% ± 6.5%) (*p* = 0.02) and Fig. 5f).

**Fig. 6:**
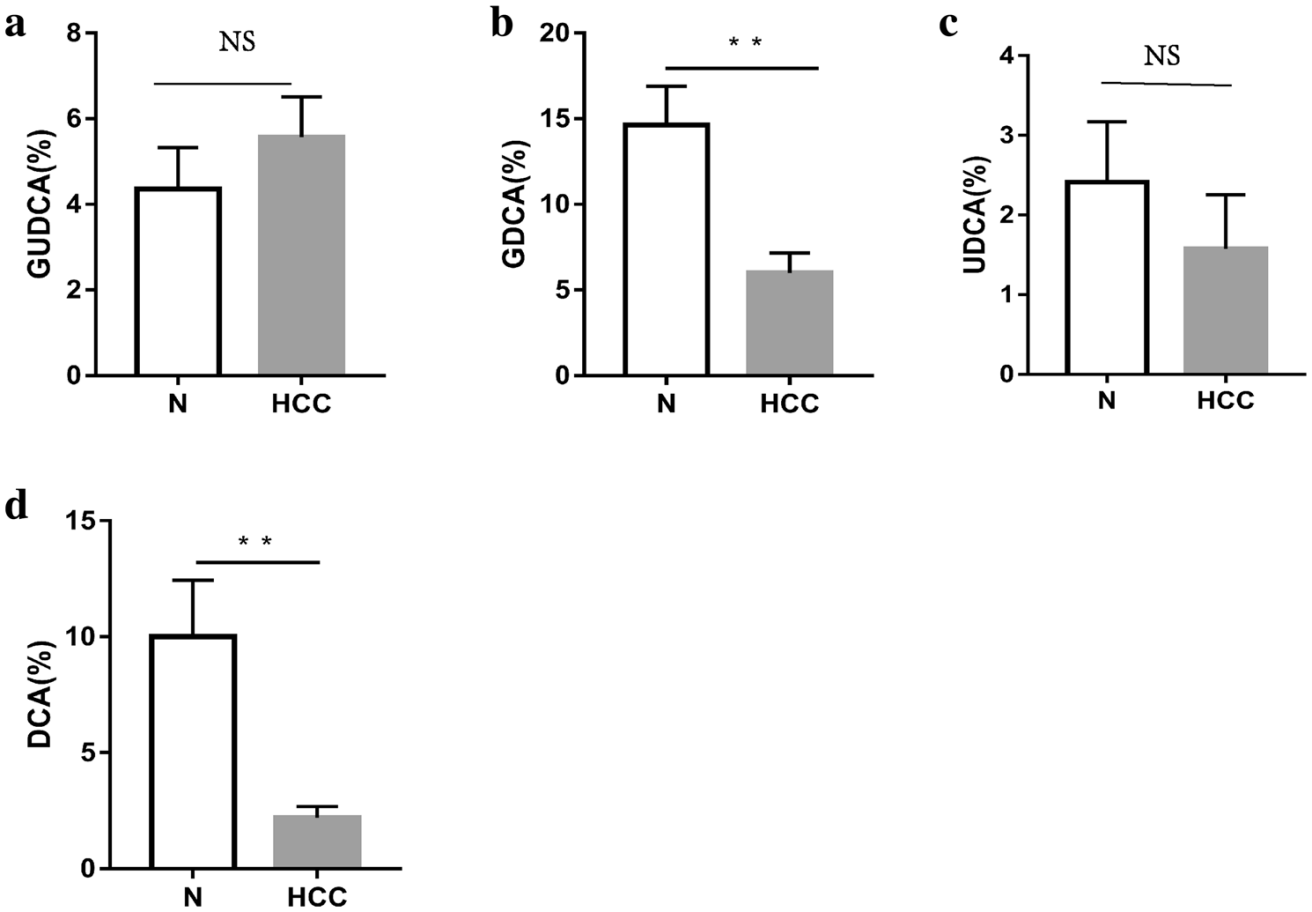
Percent of secondary bile acids in the serum of patients with HCC and healthy controls. **a**–**d** Percent of GUDCA, GDCA, UDCA, and DCA in the serum of patients with HCC and healthy controls. *HCC* Hepatocellular Carcinoma; *N* healthy controls; *p < 0.05, **p < 0.01, ***p < 0.001

**Figure7:**
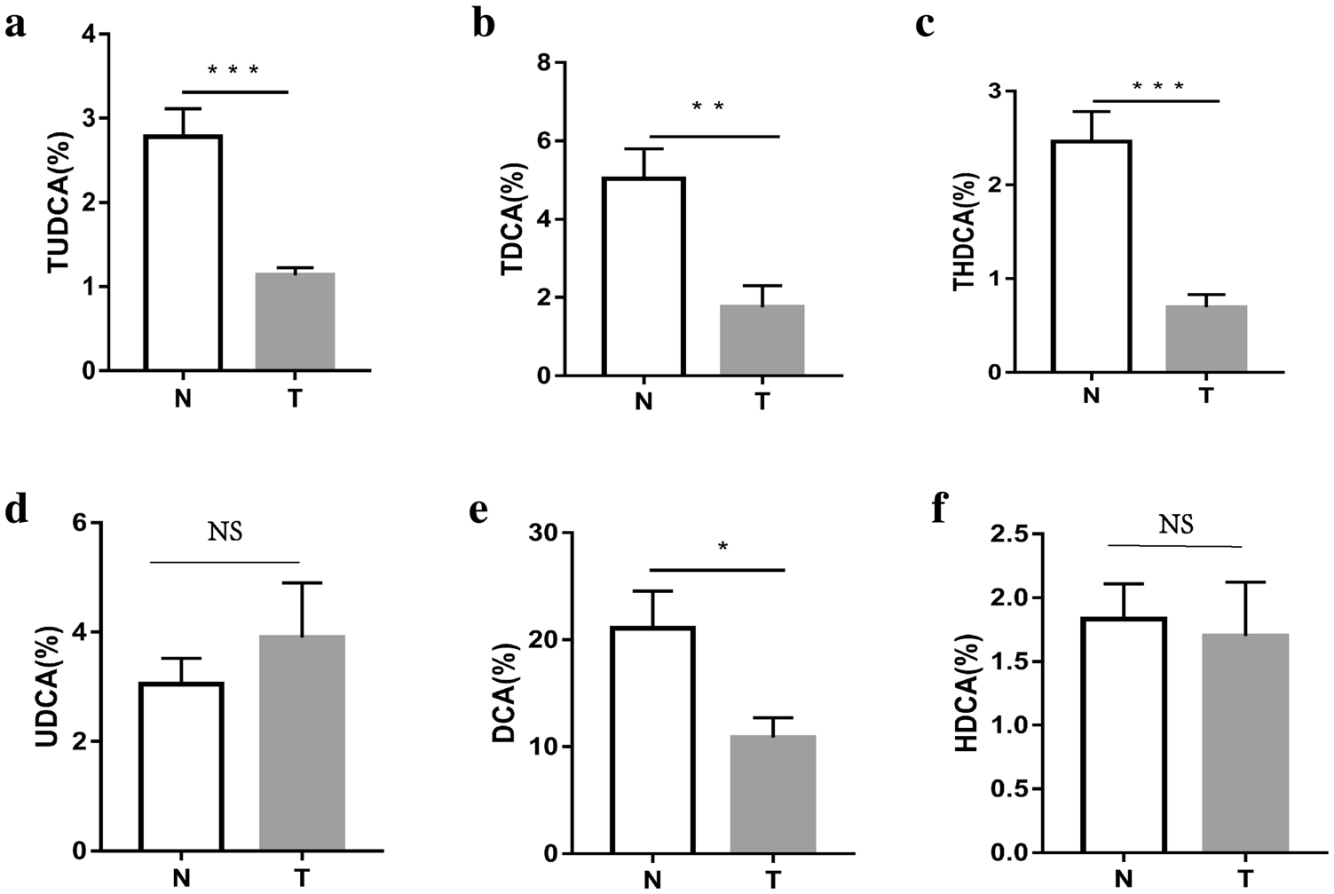
Percent of secondary bile acids in the serum of DEN-induced mice with HCC and normal control mice. **a**–**d** Percent of TUDCA, UDCA, TDCA, DCA, THDCA and HDCA in the serum DEN-induced mice with HCC and normal control mice. *T* Chemical-induced HCC mice; *N* normal control mice; *p < 0.05, **p < 0.01, ***p < 0.001

**Fig. 8:**
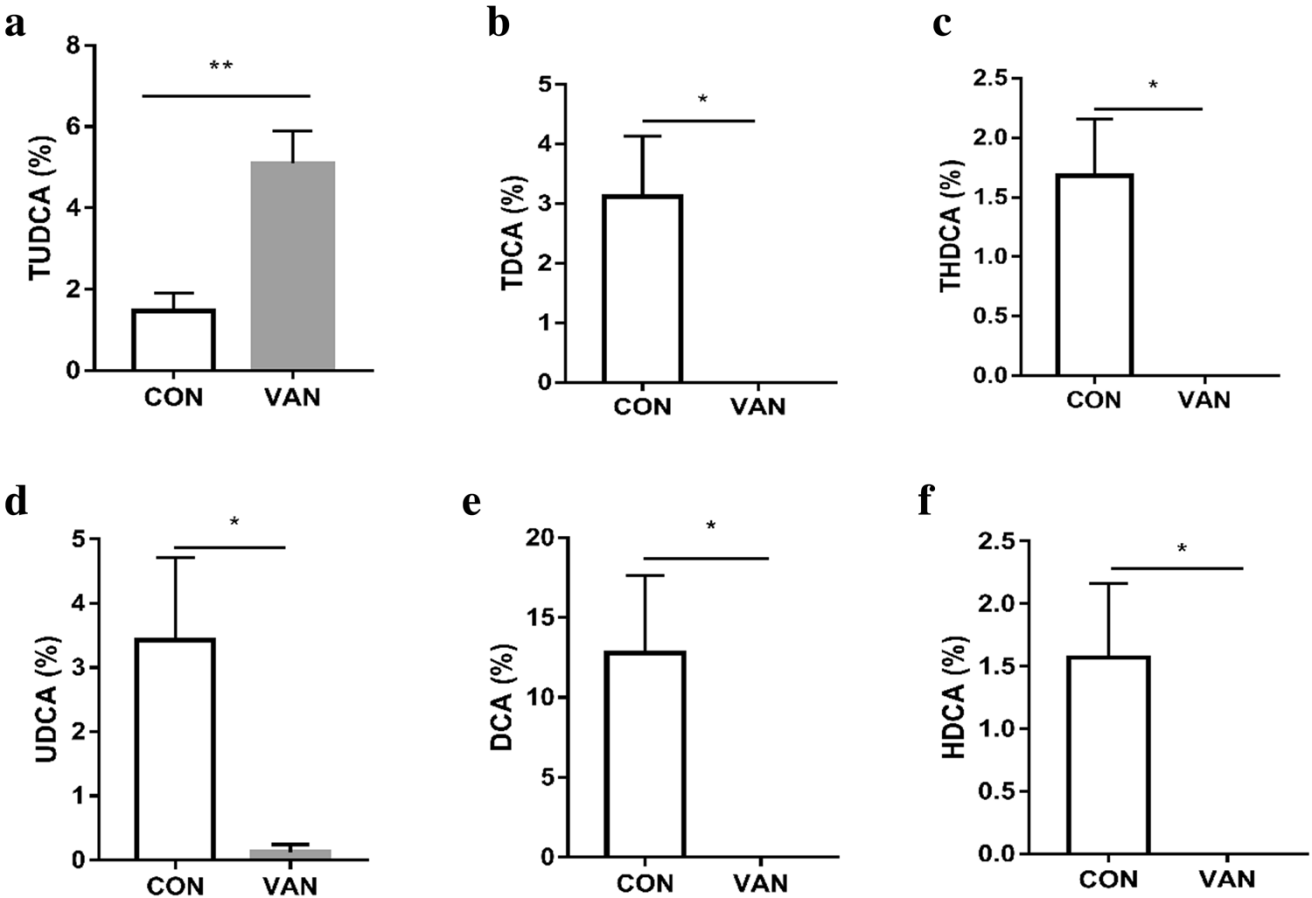
Secondary bile acids in the serum of vancomycin treatment group mice and control group mice. **a**–**f** Secondary bile acids (TUDCA, UDCA, TDCA, DCA, THDCA and HDCA) in the serum of vancomycin treatment group mice and control group mice. *VAN* vancomycin treatment group mice; *CON* control group mice; *p < 0.05, **p < 0.01, ***p < 0.001

We found that the percentages of GDCA and DCA in HCC patients were decreased significantly (Fig. 6a–d). Similarly, in the DEN-induced HCC mice, the percentages of TUDCA, TDCA, DCA, and THDCA were all significantly reduced. (Fig. 7a–f). Further analysis found that the percentages of UDCA, TDCA, DCA, THDCA and HDCA in the vancomycin treatment group were significantly reduced (Fig. 8a–f). Through the above data, we observed a remarkable reduction of serum conjugated DCA (a kind of conjugated secondary BAs) in HCC patients, DEN-induced HCC mice and vancomycin-treated mice.

### GDCA inhibits HCC growth in vivo and in vitro

**Fig. 9:**
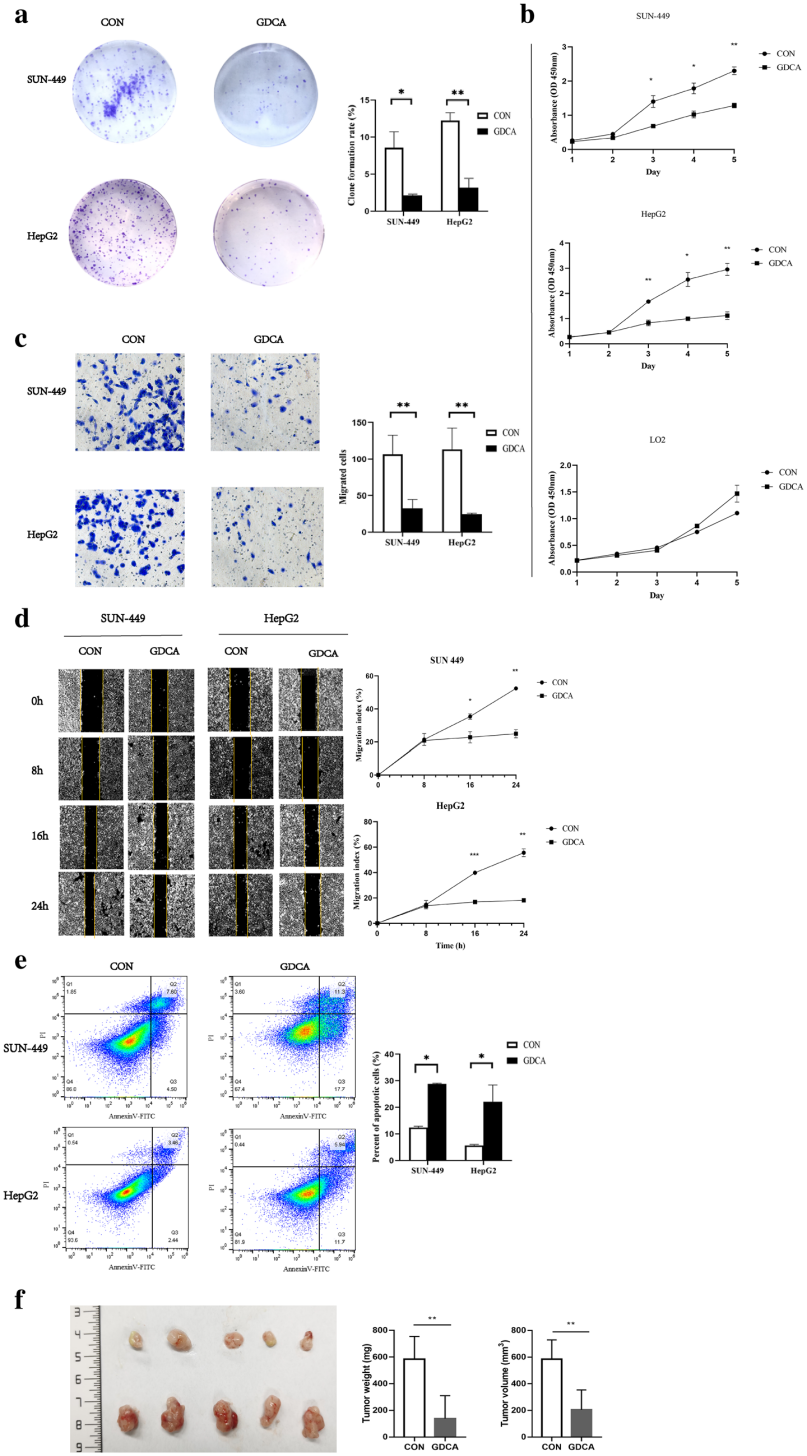
GDCA inhibits the growth and migration of HCC cells. **e** The cells were treated with GDCA (500 μm). **a** Cell proliferation were determined by Clonogenic assays. **b** Cell proliferation were determined by CCK-8. **c** Migration of HCC cells were determined by Wound Healing. **d** Migration of HCC cells were determined by Transwell assay. **e** Apoptosis of HCC cells determined by Annexin V tests. **f** Images of tumors from two groups at the time of sacrifice, and the comparison of tumor weight & volume between two groups. GDCA, GDCA treatment group HCC nude mice; CON, control group HCC nude mice; *p < 0.05, **p < 0.01, ***p < 0.001

As a kind of common conjugated DCA in humans, GDCA was used to treat human HCC cell lines, including SUN-449 and HepG2 cell lines. We found that GDCA markedly decreased the clone formation rates of SUN-449 and HepG2 cell lines compared with LO2 human hepatocyte cell lines (Fig. 9a). Using CCK-8 assays, we similarly demonstrated that the proliferation of SUN-449 cells and HepG2 cells was significantly inhibited 3 d, 4 d, and 5 d after GDCA treatment. The inhibitory rates of SUN-449 cells were 51.4%, 42.7%, and 44.1%, respectively. The inhibitory rates of HepG2 cells reached 50.9%, 66.3%, and 64.4%. However, the growth of LO2 cells was not inhibited by GDCA (Fig. 9b). Next, we studied the effect of GDCA on the migration of SUN-449 cells and HepG2 cells by Transwell assays and wound healing assays and found that GDCA significantly blocked the ability of SUN-449 cells and HepG2 cells to migrate through the membrane and refill an empty area (“scratch”, Fig. 9c, d). In addition, we used Annexin V tests to examine the effect of GDCA on the apoptosis of HCC cells (SUN-449 cells and HepG2 cells) and observed that after GDCA treatment, the apoptosis rates of SUN-449 cells (control 12.4% ± 0.46%, GDCA 28.8% ± 0.28%), (*p* = 0.013) and HepG2 cells (control 5.6% ± 0.37%, GDCA 22.1% ± 6.32%) (*p* = 0.014) were remarkably increased (Fig. 9e). We examined whether GDCA could inhibits HCC growth in HCC nude mice. We found that the tumor weight (control 617.8 ± 63.1, GDCA 179.2 ± 69.3) (*p* = 0.002) and tumor volume (control 582 ± 71.91, GDCA 241.7 ± 53.63) (*p* = 0.005) were significantly decreased after the treatment with 200 mg/kg GDCA once a day for 1 month (Fig. 9f).

## Discussion

As the main components of bile, BAs aid the digestion and absorption of fats, cholesterol, and fat-soluble vitamins from the intestinal lumen and simultaneously play an important role in stimulating hepatic bile flow and biliary excretion [28]. As amphipathic steroid molecules, BAs are synthesized from cholesterol in the liver and comprise primary BAs (CA and CDCA) and secondary BAs (DCA, LCA, and UDCA) [17]. In mice, CDCA is further converted to muricholic acid (MCA) and therefore the murine primary BAs are CA and MCA(α-MCA and β-MCA) [20]. In addition, most BAs are conjugated to glycine (predominantly in humans) or taurine (predominantly in mice) to decrease their pKa and enhance their solubility, which facilitates micelle formation in the duodenum [29, 30].

Usually, BAs are emptied into the small intestine with the gallbladder contracting after eating and function in the emulsification and absorption of lipids. In the terminal ileum, both conjugated and unconjugated BAs are almost completely (approximately 95%) reabsorbed into the portal vein by an active uptake mechanism [17, 31]. In addition, a small portion of primary BAs is readily deconjugated and 7-alpha-dehydroxylated by the microbiome to secondary BAs in the distal small intestine and colon to escape reabsorption. During the process, deconjugation by bile salt hydrolase (BSH) is a prerequisite for downstream modifications by 7-alpha-dehydroxylase or 7-alpha-hydroxysteroid dehydrogenase (HSDH) to produce DCA, LCA or UDCA. In mice tauro-conjugated CA and CDCA are deconjugated via bile salt hydrolases (BSH) and 7α‑dehydroxylated to form secondary BAs (DCA and LCA). T α-MCA and T β-MCA are deconjugated via BSH to form α-MCA and β-MCA. β-MCA is C-6 epimerized to form ω-MCA, and then ω-MCA is 7α -dehydroxylated to form hyodeoxycholic acid (HDCA). Finally, these secondary BAs can be reabsorbed passively to constitute a portion of the total BA pool, which is involved in the enterohepatic circulation, or be excreted in the feces, for example, most LCAs [16, 17]. It is well known that hydrophobic TCA, LCA, DCA and CDCA are cytotoxic, while hydrophilic BAs are cytoprotective, such as UDCA and TUDCA [18]. Therefore, we focused on hydrophilic secondary BAs and conjugated secondary BAs. In humans, secondary BAs are mainly composed of GUDCA, UDCA, GDCA and DCA. In mice, secondary BAs include TUDCA, UDCA, TDCA, DCA, THDCA and HDCA.

Besides facilitating lipid absorption, BAs have emerged as relevant signaling molecules to activate bile acid receptors, such as farnesoid X receptor (FXR, also known as NR1H4) and G protein-coupled bile acid receptor 1 (TGR5, also known as GPBAR1), and to regulate their own synthesis as well as other metabolic processes, such as glucose, lipid, and energy homeostasis [31]. Cariou et al. found that serum concentrations of CDCA, CA, and DCA in humans are positively related to insulin resistance [32]. Jiao et al. indicated that serum levels of total BAs were increased in patients with NAFlD, and the percentage of FXR antagonistic DCA was increased, while the percentage of its agonistic CDCA was decreased in NAFlD [33]. Xie et al. indicated that the hepatic concentrations of TCA, GCA, TCDCA, DCA, TDCA, TUDCA, TLCA and total BAs were substantially increased in the fibrosis phase of mice (weeks 12 and 20), while fecal BAs were decreased at the same stage [22]. Furthermore, Allen et al. found that DCA induced the expression of inflammatory genes in hepatocytes, which is closely associated with the development of cancer [5]. However, we found few studies on the role of bile acids in HCC patients.

In our study, we first found that the proportion of serum secondary BAs in HCC patients was significantly lower than that in normal healthy persons, although the levels of their total serum BAs were similar. Furthermore, we generated a HCC mouse model using DEN and CCl4 and observed that the serum levels of total bile acids were markedly increased, while the proportion of secondary BAs was significantly decreased in mice with HCC. Similarly, Xie et al. also found that the primary BAs TCA and TCDCA were increased in the plasma and liver of mice at the HCC stage compared with those in normal controls [22]. Moreover, cholestyramine treatment enhanced the intestinal excretion of hydrophobic BAs to prevent HCC development in mouse models. Likewise, Kakiyama et al. showed that in the data of patients with advanced cirrhosis, the levels of serum primary BAs in the patients with advanced cirrhosis were significantly increased [34]. Therefore, we can deduce that the decrease in the serum secondary BA rate may be closely related to HCC development.

Given that the gut microbiota can influence the size and composition of the BA pool by deconjugation and downstream modifications, we further observed the composition of the gut microbiota in HCC patients and HCC mouse models and probed the roles of bile acid–microbiota crosstalk in HCC development. We found a remarkable difference between the gut bacteria of HCC patients and healthy controls. Beta diversity by PCoA indicated the different distributions of the fecal microbial community between the two groups. A Venn diagram also showed that HCC patients had larger and more unique gut bacterial community than normal healthy controls. This finding is similar to previous studies, which indicated greater richness or diversity in the bacterial community likely suggested the overgrowth of various harmful bacteria in patients with HCC [11]. Then, we further analyzed the composition of the fecal microbiota of HCC patients and healthy persons at the phylum or order levels and observed that Bacteroidetes, Firmicutes, Proteobacteria, and Actinobacteria together accounted for up to 90% of sequences on average; in particular, the phylum Actinobacteria and order Bifidobacteriales were both markedly depleted in patients with HCC. This is also coincident with the results of other studies. For example, Zhang et al. indicated that the depletion of Bifidobacterium in the gut microbiome was observed in mice fed high dietary cholesterol, which was confirmed in patients with hypercholesteremia. In addition, dietary cholesterol drives the formation of NAFLD-related HCC in mice [35, 36]. Ponziani et al. also showed the enrichment of Bacteroides and Ruminococcaceae and the reduction of Bifidobacterium in patients with NAFLD-related HCC [37]. In the DEN-induced HCC mice, the gut bacterial composition was also significantly different from that of healthy control mice, and there was a larger abundance and more unique bacteria. However, the top ten bacteria in mice were not completely the same as those in humans, although Bacteroidetes, Firmicutes, Proteobacteria, and Actinobacteria together still accounted for up to 90% of sequences on an average. At the order level, we found a marked increase in Erysipelotrichales and Coriobacteriales and a reduction in Clostridiales and Desulfovibrionales in HCC mice. Given that secondary BAs are converted through deconjugation via bile salt hydrolases (BSH), our further study focused on the abundance of BSH-rich bacteria (Bifidobacteriales, Lactobacillales, Bacteroidales, and Clostridiales) [33] and found that it was significantly decreased in DEN-induced HCC mice, although it was slightly lower in HCC patients than in healthy persons. Therefore, we assume that the reduction in BSH-rich bacteria may result in the lowering of secondary BAs, which is closely associated with the development of HCC.

To further confirm our deduction, we induced a decrease in BSH-rich bacteria in tumor-bearing mice with vancomycin treatment and observed larger HCC xenografts. Meanwhile, a reduction in the secondary BA rate was observed after vancomycin treatment. Therefore, we can conclude that BSH-rich bacteria may induce an increase in the serum level of secondary BAs, which are involved in the inhibition of HCC. It is well known that secondary BAs are comprised of unconjugated and conjugated BAs, and much evidence indicates that hydrophobic BAs, including the unconjugated secondary BAs LCA and DCA [5], can directly damage liver cell membranes and induce cell apoptosis, which releases inflammatory factors and promotes the development of HCC. In addition, Wu et al. indicated that the hydrophilic conjugated secondary BA TUDCA prevented Mst1/2 mutant-driven hepatic carcinogenesis by inhibiting the activation of the Yap pathway and attenuating the unfolded protein response (UPR) in mice [21]. Actually, UDCA and its amidated conjugate TUDCA have been widely used as therapeutic drugs for patients with cholestatic liver diseases. Their protective mechanisms are considered to be related to preventing the formation of ROS, inhibiting the translocation of the pro-apoptotic protein Bax from the cytosol to the mitochondria, and even directly stabilizing mitochondrial membranes [31]. Therefore, we focused on conjugated secondary BAs and analyzed the specific components of conjugated secondary BAs associated with the development of HCC. We found that the percentages of GDCA in HCC patients were significantly decreased, the percentages of TUDCA, TDCA and THDCA were all significantly decreased in DEN-induced HCC mice, and the percentages of UDCA, TDCA, and THDCA were significantly decreased in vancomycin-treated mice with HCC. Therefore, the percentage of conjugated DCA (GDCA and TDCA) was found to be intimately associated with HCC from humans or mice.

Whether do these conjugated DCA influence the growth of HCC ? To answer this question, we used GDCA to treat human HCC cell lines (SUN-449 and HepG2) and found that GDCA remarkably inhibited the proliferation and migration of HCC cells as well as promoted the apoptosis of HCC cells without affecting the growth of LO2 human hepatocytes. In vivo, the treatment with 200 mg/kg GDCA significantly inhibited the growth of HCC tumors.

In summary, our results show that the bile acid profile of HCC is characterized by the remarkable decrease in protective secondary BAs, in particular, conjugated DCA, which may be closely associated with the reduction in BSH-rich bacteria in the gut, in particular probiotic Bifidobacteriales in humans. The mechanisms may be correlated with conjugated DCA directly inhibiting the growth and migration of HCC cells. In the future, it is necessary and valuable to further explore the specific roles and mechanisms of gut microbiota-bile acid crosstalk in the occurrence and development of HCC, and to exploit effective treatments for HCC. For example, we will use humanized-liver mice or knockout mice, and make several animal models with different treatments of bacteria or bile acids. Even, the further studies should be done to explore the effects and molecular mechanisms of bacteria or bile acids on tumor microenvironment.

## Supplementary Information

Below is the link to the electronic supplementary material.

Supplementary file1 (DOC 744 KB)

## Data Availability

The data that support this study are available on request from the corresponding author. The data are not publicly available due to privacy or ethical restrictions.

## Abbreviations

HCC: Hepatocellular carcinoma
BAs: Bile acids
UHPLC-MS/MS: Ultra-high performance liquid chromatography tandem mass spectrometry
BSH: Bile salt hydrolase
DEN-HCCmice: Chemical-induced HCC mouse models
Vancomycin-treated mice: Mouse orthotopic implanted liver tumor models
DEN: Diethylnitrosamine
LCA: Lithocholic acid
UDCA: Ursodeoxycholic acid
HDCA: Hyodeoxycholic acid
CDCA: Chenodeoxycholic acid
DCA: Deoxycholic acid
α-MCA: α-Muricholic acid
β-MCA: β-Muricholic acid
CA: Cholic acid
GUDCA: Glycoursodeoxycholic acid
GCDCA: Glycochenodeoxycholic acid
GDCA: Glycodeoxycholic acid
GCA: Glycocholic acid
TUDCA: Tauroursodeoxycholic acid
THDCA: Taurohyodeoxycholic acid
TCDCA: Taurochenodeoxycholic acid
TDCA: Taurodeoxycholic acid
T-α-MCA: Tauro α-Muricholic acid
T-β-MCA: Tauro β-Muricholic acid
TCA: Taurocholic acid
HSDH: 7-Alpha-hydroxysteroid dehydrogenase
FXR: Farnesoid X receptor
TGR5: G Protein­coupled bile acid receptor 1
