# Supplementary material for: Abnormal bile acid-microbiota crosstalk promotes the development of hepatocellular carcinoma
Source: Hepatol Int. 2022 Feb 24;16(2):396–411. doi: 10.1007/s12072-022-10299-7 (PMC9013324; doi:10.1007/s12072-022-10299-7)

**Original photo**


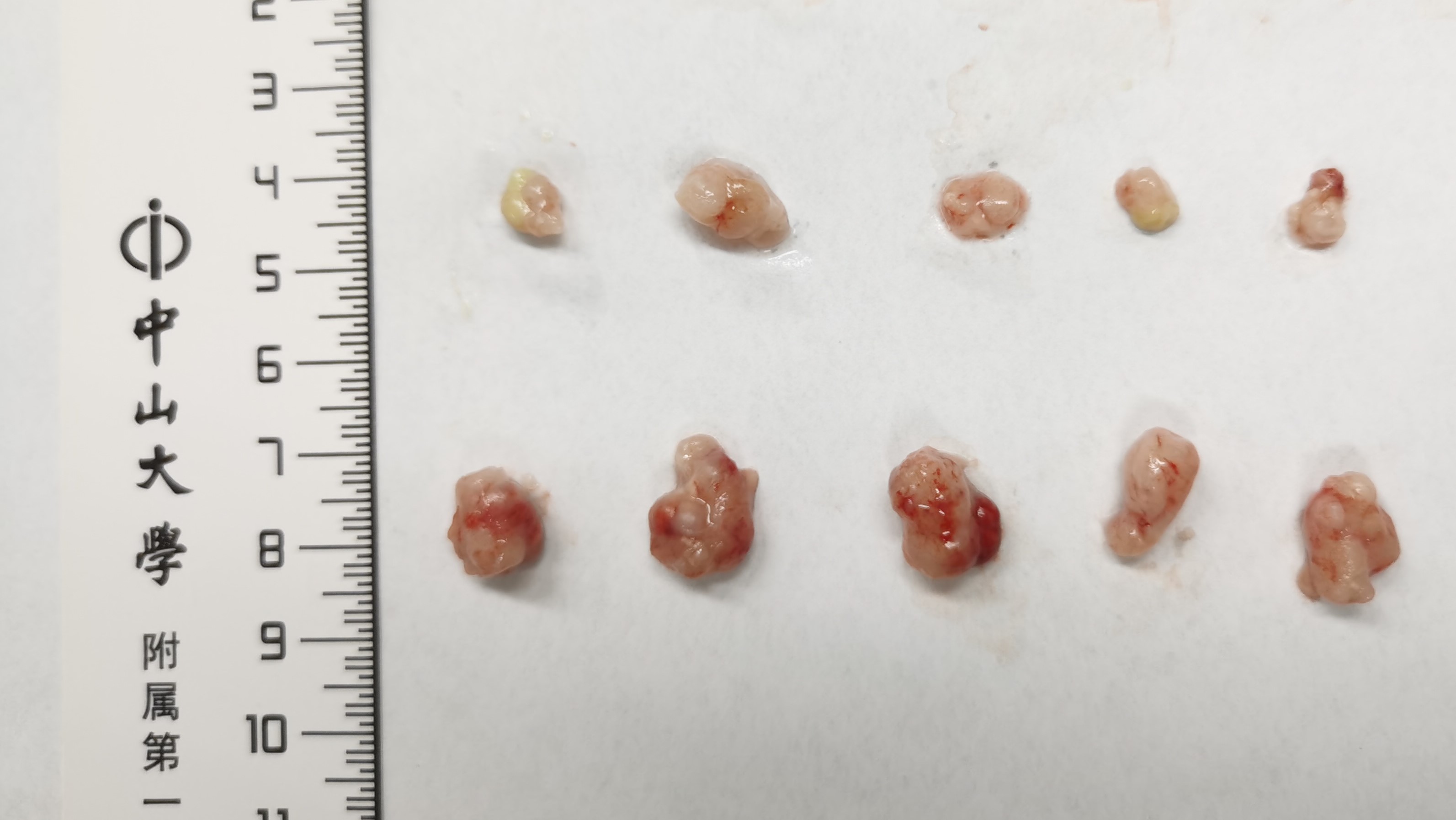


8 week-old

Nude mice

Injection

SUN-449

**day10 GDCA 200mg/Kg**

**day7 day14 day21 day28 day35 day42 sacrificed**

**Raw data of nude mice**


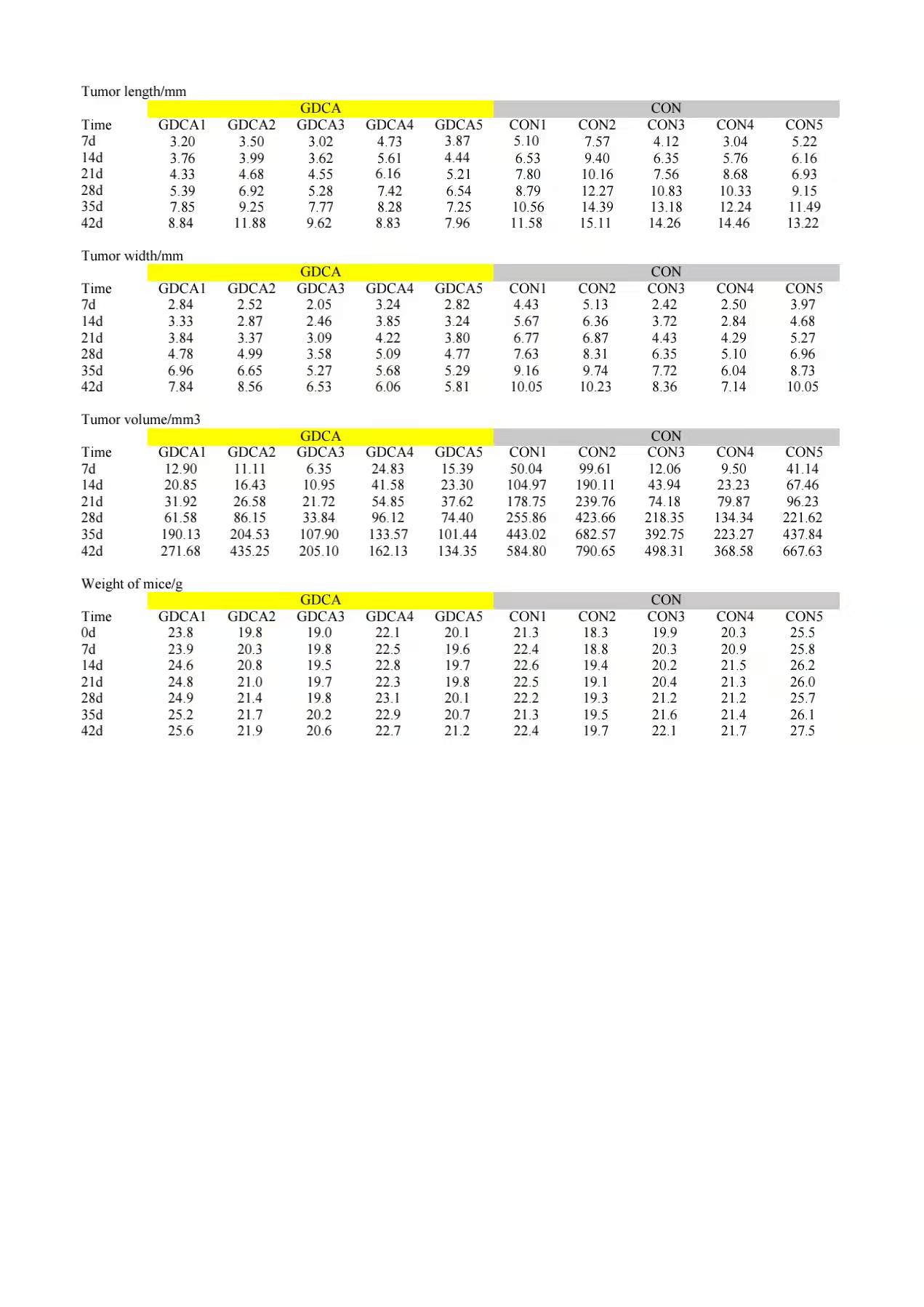

Supplement: Supplementary file 1 — Supplementary file1 (DOC 744 KB) [file 12072_2022_10299_MOESM1_ESM.doc]
